# Radiographic Assessment of the Technical Quality and Periapical Health of Root-Filled Teeth Performed by General Practitioners in a Turkish Subpopulation

**DOI:** 10.1155/2013/514841

**Published:** 2013-02-02

**Authors:** E. Tarim Ertas, H. Ertas, Y. Sisman, B. Sagsen, O. Er

**Affiliations:** ^1^Department of Oral and Maxillofacial Radiology, Faculty of Dentistry, Izmir Katip Celebi University, Izmir 35640, Turkey; ^2^Department of Endodontics, Faculty of Dentistry, Izmir Katip Celebi University, Izmir, Turkey; ^3^Department of Oral and Maxillofacial Radiology, Faculty of Dentistry, Erciyes University, Kayseri, Turkey; ^4^Department of Endodontics, Faculty of Dentistry, Erciyes University, Kayseri, Turkey

## Abstract

*Aim*. The aim of this study was to evaluate by means of radiographs the technical quality of root fillings performed by dental practitioners. *Methods and Material*. Standardized periapical radiographs were made on 484 patients who received endodontic treatment in private practice. A total of 831 endodontically treated teeth with 1448 roots were evaluated for technical quality of the root canal filling and the periapical status of the teeth. Also, the apical status of each root-filled tooth was assessed according to the length, density, and taper of root fillings, and the presence of apical transportation, broken root instruments, and overfilled sealer or gutta-percha material was recorded for each root canal. *Results*. Of the endodontically treated teeth 26.6% had healthy periapical tissues, while technically good endodontic treatment constituted 12.8%. Based on the treatment success, there was no significant difference between the tooth groups. Statistical analysis of the data did not demonstrate statistically significant differences between the various parameters that were evaluated (*P* < 0.05). *Conclusions*. Technical quality of root fillings in a population who received treatment in private practice was poor and was consistent with a low prevalence of apical health. The probable reasons for this failure are multifactorial and may be improved if the operators improve their skills with continuing postgraduate education programs.

## 1. Introduction

In many countries, dental health is a rising and important public health problem because of its medical, economic, and ethical repercussions [1], in which root canal treatment (RCT) plays an essential role when considering the quality of dental care [2, 3]. With the improvement of modern principles in endodontic practice, many clinical studies have shown an increase in the favorable treatment outcomes [4–6]. Epidemiological studies evaluating the outcome of the endodontic treatment performed in controlled clinical environments (e.g., dental schools) have demonstrated success rates up to 95% [4, 7, 8]. Concerning the recommendations of competent scientific committees, experts, and professional guidelines related to the quality of treatments, there is evidence that the quality of treatments completed in general dental practice is less than ideal [9–11]. Irrespective of the population group and geographic location, success rates decrease as low as 35% to 60% [11–15]. As a result, with a decrease in the quality of treatment, there is an increase in the prevalence of apical periodontitis, so it is necessary to improve the quality of root canal treatment in general dental practice in order to promote periradicular health [16]. 

There is a general assumption that an endodontist provides treatment of better quality and treatment outcomes compared to generalists, so the aim of this study was to assess the radiographic assessment of the technical quality and periapical health of root-filled teeth performed by general practitioners in a Turkish subpopulation.

In addition, the quality and type of coronal restorations and relation between the coronal restoration and periapical health were also evaluated.

## 2. Subjects and Methods

### 2.1. Patient Selection

This study was undertaken by evaluating the periapical status of teeth on periapical radiographs of randomly selected new patients seeking routine dental care between the ages of 9 and 71 (median 35) who first presented to the Oral Diagnosis and Radiology Department for a general dental checkup and then were referred to the Endodontics Department of the Faculty of Dentistry, Erciyes University, Kayseri, Turkey. Patients attending to the university clinic for the first time were selected, and to be included in the study the patient's chart had to contain periapical radiographs made at the Oral Diagnosis and Radiology Department of the school and depicting previous root canal treated teeth performed by outside dentists. Also, the other criteria for inclusion in the study were that the patient's chart had to contain periapical radiographs of the previously root canal treated teeth during patient examination. Since these periapical radiographs had originally been taken for definite radiological diagnosis previously, it was therefore not necessary to seek ethical approval for this study.

For the study, 1448 roots of 831 endodontically treated teeth obtained from 484 patients (335 female and 149 male) were evaluated. 

### 2.2. Radiographic Examination

All periapical radiographs were taken with a Trophy CCX X-ray unit (Trophy Radiographie-94300, Vincenners, France) using Ultra Speed film (Eastman Kodak, Roche Sister, NY, USA) and processed with an automatic film processor according to the manufacturer's instructions. The periapical radiographs were evaluated by an experienced radiologist using an illuminated viewer box with ×2 magnification.

### 2.3. Radiographic Evaluation

The radiographs were analyzed for several variables such as radiographic quality and health of the apical status according to the criteria proposed by De Moor et al. [11] (Table 1).

The criteria proposed by Siqueira et al. [17] were used in the evaluation of the coronal restorations (Table 1). 

Besides the parameters above, if complications such as lateral or furcal perforations and fractured instruments were detected, the root filling was considered as inadequate.

### 2.4. Statistical Analysis

Evaluations of the teeth and the roots were analyzed using SPSS software, Version 16.0 (SPSS Inc., Chicago, IL, USA). The influence of the endodontic treatments, coronal restorations, existing complications, including other possible combinations on the periapical health were analyzed using the chi-square test. *P* < 0.05 values were considered as statistically significant.

## 3. Results

In the study, 1448 roots of 831 teeth obtained from 484 patients (with a median age of 35 years) were evaluated. The root-filled teeth belonging to females comprised 68.8% of the study group whereas those of the males comprised 31.2%.

The types of teeth examined in this study are shown in Table 2. Four hundred eighty-seven were maxillary teeth (58.6%) and 344 were mandibular teeth (41.4%). Of 831 root-filled teeth, molars were the most frequently treated teeth (*n* = 343), followed by premolars (*n* = 282), incisors (*n* = 136), and canines (*n* = 70).

The periapical status of all root-treated teeth is presented in Table 2. Of the 831 root-filled teeth investigated in the study, only 26.6% of teeth had healthy periapical structure. The relationship between the periapical status and the gender was not statistically significant (*P* > 0.05); the distribution is presented in Table 2. 

The relationship between the periodontal health and distribution of teeth in dental arches is shown in Table 2, showing that 371 maxillary and 239 mandibular teeth had apical periodontitis, while the apical periodontium of only 116 maxillary and 105 mandibular teeth was healthy.

Of 831 root-treated teeth, 613 teeth (73.8%) had inadequate length of filling, being short of the radiographic apex or overfilled, whereas only 218 (26.2%) had ideal filling length. The relationship between the periapical health status and the length of fillings is shown in Table 2.

As another evaluation criteria of root fillings, the smoothness and continuity of the taper were examined and only 259 teeth (31.2%) were recorded as having ideal taper while 572 teeth (68.8%) had inadequate taper (Table 2). There was a statistically significant relationship between the taper and periapical health in our study sample (*P* < 0.001). 

In addition, 57.9% (481) of all root-treated teeth had inadequate density of filling, while 42.1% (350) had adequate density.

A root canal filling was considered adequate only when the length, tapering, and density of the filling were acceptable. Taking these criteria together, adequate root canal fillings were found in only 12.8% of all examined samples. 

Overall, when the coronal structure of the teeth with root fillings was evaluated, 34.4% of all had crown restoration. While 28.2% of all root canal-treated teeth had poor or missing intracoronal restorations, only 37.4% were recorded with adequate intracoronal restorations (Table 2). 

In the present study, without considering the technical quality of root canal treatments, there is a statistically significant difference between the prevalence of apical health of the teeth with poor (19%) and adequate (32%) coronal restorations (*P* < 0.001). 

Finally, when we looked for the existing complications, 49 teeth had been overfilled with gutta-percha or sealer material, 30 teeth had broken instruments inside the canals, and 16 teeth had apical transportation.

## 4. Discussion

The population investigated in this study consisted of adult patients attending the Dental School of Erciyes University, Kayseri, Turkey, for the first time for a periodic checkup or required general dental treatment. For the investigation, we arranged a cross-sectional study in order to reveal the situation of endodontic treatments, the quality of the performed treatments, and the periapical status of the study population, as applied by general dental practitioners. 

As a routine examination and diagnosis protocol, we take panoramic radiographs from most of our patients for a detailed evaluation since all teeth can be seen on one radiograph and deliver a relatively low patient radiation dose when compared with full-mouth sets of periapical radiographs. Although panoramic radiographs are accepted to be sensitive in the diagnosis of periapical pathosis [18], the accuracy of panoramic radiographs in the detection of apical periodontitis has been questioned [19]. Additionally, because of nonuniform magnification and poor imaging of anterior teeth, we did not find panoramic radiographs sufficient and took periapical radiographs of the suspected teeth with the approval of the patients after explaining the necessity, so periapical radiographs were used instead of panoramic radiographs [20]. Also, greater interobserver variability with panoramic radiographs [2] is another reason for our choice of periapical radiographs. 

A main disadvantage of this cross-sectional study is that the radiographs were examined at a given point in time, and no information was available as to the time passed since endodontic treatment, so it is impossible to determine whether a periapical lesion is healing or not [21]. Because a radiograph provides only static information of a dynamic process, it is difficult to guess if a periapical lesion is increasing or decreasing in size [22]. In order to overcome these kinds of disadvantages of the present study, patients were asked to provide the time endodontic treatments or restorations were performed, the clinician who performed the work, and the clinician's level of training, and the time passed between the treatment and the observation. According to the answers, the patients who could not know for certain about the questions, or whose treatment was not performed by a general dental practitioner, or who had had root canal treatment within 2 years were excluded from the study. 

In the literature, many epidemiological studies have shown that, because of the poor technical quality of root fillings, there is a high prevalence of periapical disease in these teeth. In these studies, there is a wide range of percentage of inadequate root fillings reported, between 49% and 87%, many of which were undertaken in general practice or hospital settings [23]. For several years, most studies reported in the literature have shown that the technical quality of root canal fillings performed by general dental practitioners is often less than ideal [10, 11, 24, 25]. One of the numerous possible reasons for this may be the withdrawal of dental practitioners from many of the principles and techniques of endodontics that were covered at dental schools [26, 27] and/or the reported adaptation of many newer techniques that have little scientific basis [27]. A study performed in the northeast of England reported that general dental practitioners felt they needed more continuing education and postgraduate courses to improve their clinical skills in endodontics [28]. On the contrary, results of the studies carried out in controlled environments have shown that, with higher technical standards of fillings, the presence of disease appears to have a minimal influence on the outcome of the treatment. Besides, the survival rate of teeth treated by endodontists was shown to be significantly better than those treated by general practitioners [29]. In their study, Ingle et al. demonstrated that highly skilled operators are less likely to perform procedural errors that may ultimately compromise the prognosis [30].

Our study group consisted of 69.2% females and 30.8% males (*P* < 0.001). In some epidemiological studies, gender was reported to have no effect on the number of roots filled or the presence of apical periodontitis [15, 31, 32]. Our results related to the female prominence were similar to those reported by Georgopoulou et al. [33] and Sunay et al. [19], which may indicate the greater interest of female patents in dental care and attendance for checkups [19]. 

Maxillary and mandibular molars, followed by premolars were mostly treated with root canal treatment in our study sample. Compared with mandibulars, maxillary teeth had a higher prevalence of apical periodontitis. Because maxillary molars may exhibit many morphological variations of their complex root canal anatomies, this may be the reason for the higher prevalence of failure performed in these teeth [34]. In addition, because of the complex anatomy of the maxillary and zygomatic bones, the difficulty of radiographing the maxillary teeth during treatment and generally the poorer radiographic quality may be the other reasons of the failure of these treatments. 

In the present study, 73.4% of the root-filled teeth were associated with periapical lesions and only 26.6% of teeth had healthy periapical structure. This high percentage was certainly a result of the high frequency of inadequate endodontic treatment in the investigated group, as it is generally accepted that the quality of the endodontic treatment strongly influences the status of the periradicular tissues [4, 11, 12, 15].

In our study, a radiographic ruler as a measuring tool was used to allow an accurate measurement of the proximity of the root canal filling relative to the radiographic apex. The percentage of root fillings with adequate length was 26.20% in the present study. In the literature, many studies have considered the ideal length of filling within 2 mm of the radiographic apex as the gold standard [10, 35, 36], and in Sjögren et al. [4] in their highly controlled long-term outcome study, the investigators reported the close relation of healing of apical periodontitis (94%) and the roots filled ideally close (0–2 mm) to apex [23]. In the same study, the healing rates of apical periodontitis of short filled teeth were 68%, while the rate was 76% with the overfilled teeth.

As another evaluation criterion of root fillings, the prevalence of the smoothness and continuity of the taper in the present study was 31.2% while the remainder had 68.8% of inadequate taper. Because the taper of root canals is a more subjective criterion, in the literature only a few reports have been published on this matter [37]. In a recent study performed by Bierenkrant et al. [23], the authors reported the majority of the canals had a “smooth and continuous” taper of 83.8% prevalence rate [23]. The same authors correlated their high rate with the benefit of rotary NiTi instrumentation [23]. As the European Society of Endodontology (1994) advises the preparation of root canals should be tapered from crown to apex, we believe in the future, with the use of NiTi rotary instruments, the philosophies of both instrumentation and filling of the root canals will be changed in a positive manner. Additionally, we found a statistically significant relationship between the taper and periapical health in our study sample (*P* < 0.001).

In the literature, there is conflicting evidence related to the impact of root-filling density on prognosis [23]. Some studies have shown that teeth with homogenous root fillings will result in more consistent healing [7] and survival [38], whereas some of the other studies reported no difference in the prognosis of root canal treatment [4], and some reported a significantly increasing prevalence of apical periodontitis with nonhomogenous and inadequately compacted root fillings [16, 39, 40]. In the present study, 57.9% (481) of all root-treated teeth had inadequate density of filling, while 42.1% (350) had adequate filling density.

A root canal filling was considered adequate only when the length, tapering, and density of the filling were acceptable. With these criteria together, adequate root canal fillings were found in only 12.8% of all examined samples. Unfortunately, this prevalence result is the lowest of all reviewed articles published in the literature, among which was reported an acceptable root filling in the range of 17.7–64.5% [23, 41]. When considering the treatment adequacy between the tooth types, there was no statistically significant difference in our study sample (*P* > 0.05).

The quality of the coronal seal is another important factor that influences the periapical health [41]. In many studies, the association between the quality of the coronal seal and the presence of periapical radiolucencies were shown in a negative correlation [21, 41–43]. In the present study, when the coronal structure of the root canal-treated teeth was evaluated, 34.4% of all had crown restoration. While 28.2% of all root canal-treated teeth had poor or missing intracoronal restorations, only 37.4% were recorded with adequate intracoronal restorations (Table 2). 

In the present study, teeth without or inadequate coronal restoration had significantly more periapical pathology compared to those with adequate coronal restorations (Table 2). Without considering the technical quality of root canal treatments, there is a statistically significant difference between the prevalence of apical health of the teeth with poor (19%) and adequate (32%) coronal restorations (*P* < 0.001). 

Ingle et al. [30] demonstrated in their study that highly skilled operators are less likely to perform procedural errors that may ultimately compromise the prognosis. For the present study, 49 teeth had overfilled gutta-percha or sealer material, 30 teeth had broken instruments inside the canals, and 16 teeth had apical transportation. It is clear that the operator skill and the condition under which the treatment was delivered are important factors that should not be underestimated on the prognosis of the treatments [18]. 

## 5. Conclusion

Within the limitations of this study, the technical quality of root filling performed by dental practitioners in Kayseri, Turkey, is very low compared to other studies. The probable reasons for this failure are multifactorial and may be improved if the operators improve their skills with continuing postgraduate education programs. 

## Figures and Tables

**Table 1:** Parameters recorded on endodontically treated teeth.

Parameters	Criteria	Definition
Quality of root canal treatments		
Length of root canal filling	Adequate	Root filling ending ≤2 mm from the radiographic apex
	Poor	Root filling beyond the radiographic apex or root filling >2 mm from the radiographic apex
Density of root canal filling	Adequate	No voids present in the root filling or between root filling and root canal walls
	Poor	Voids present in the root filling or between root filling and root canal walls
Taper of root canal filling	Adequate	Consistent taper from the apex to the coronal part
	Poor	Not consistent taper from the apex to the coronal part

Periapical status		
	Healthy periodontal ligament	If the periodontal ligament was intact with no signs of periapical pathosis
	Apical periodontitis	If the widening of the apical part of the periodontal ligament was not exceeding two times the width of the lateral periodontal ligament space, teeth were catogorized as having widening of the periodontal ligament
	Apical periodontitis	If the periapical radiolucency in connection with the apical part of the tooth exceeding at least two times the width of the lateral part of the periodontal ligament, such teeth were categorized as having obvious periapical radiolucency

Condition of coronal restoration		
	Adequate	Any permanent restoration that appeared intact radiographically
	Poor	Any permanent restoration with detectable radiographic signs of overhangs, open margins, or recurrent caries, or presence of temporary coronal restorations
	Missing	No permanent or temporary coronal restoration

**Table 2:** Periapical status and related criteria.

					Periapical status		
	Total		Apical periodontitis		Healthy		Chi-square
	*n*	%	*n*	%	*n*	%	*P* values
Gender							
Female	259	31,2%	201	77,6%	58	22,4%	0.065
Male	572	68,8%	409	71,5%	163	28,5%	
Dental arche							
Maxilla	487	58,6%	371	76,2%	116	23,8%	0.031
Mandibula	344	41,4%	239	69,5%	105	30,5%	
Tooth type							
Incisors	136	16,4%	102	75,0%	34	25,0%	0.002
Canines	70	8,4%	46	65,7%	24	34,3%	
Premolars	282	33,9%	189	67,0%	93	33,0%	
Molars	343	41,3%	273	79,6%	70	20,4%	
Filling length							
Poor	613	73,8%	500	81,6%	113	18,4%	<0.001
Adequate	218	26,2%	110	50,5%	108	49,5%	
Taper							
Poor	572	68,8%	456	79,7%	116	20,3%	<0.001
Adequate	259	31,2%	154	59,5%	105	40,5%	
Filling density							
Poor	481	57,9%	401	83,4%	80	16,6%	<0.001
Adequate	350	42,1%	209	59,7%	141	40,3%	
Type and quality of coronal structure							
Inadequate filling	234	28,2%	189	80,8%	45	19,2%	<0.001
Adequate filling	311	37,4%	210	67,5%	101	32,5%	
Crowned	286	34,4%	211	73,8%	75	26,2%	
